# Screening of optimal harvesting period and drying methods for Paeoniae Radix Rubra based on eight bioactive compounds and biomass

**DOI:** 10.3389/fnut.2026.1774758

**Published:** 2026-06-16

**Authors:** Deshuai Guo, Defeng Zhuang, Junying Jia, Yuan Guo, Zhijun Li, Dezhi Sun, Xiaoze Yu

**Affiliations:** 1 College of Agronomy, Inner Mongolia Minzu University, Tongliao, China; 2 University Engineering Research Center of Chinese (Mongolia) Ecological Planting Medicinal Materials in Inner Mongolia Autonomous Region, Tongliao, China; 3 Inner Mongolia Minzu University College of Engineering, Tongliao, China

**Keywords:** CRITIC, drying methods, harvesting period, Paeoniae Radix Rubra, pharmaceutical active substances, relevance analysis

## Abstract

**Objective:**

To identify the optimal harvesting age, timing, and drying method for Paeoniae Radix Rubra in Tongliao, Inner Mongolia, China.

**Methods:**

The plant traits, biomass, and contents of eight pharmacodynamic substances were measured in 3-, 4-, and 5-year-old cultivated *Paeonia lactiflora* Pall. plants. 5 drying methods were applied to the roots of 4-year-old root. The bioactive components were quantified, followed by comprehensive evaluation using the CRITIC method and correlation analysis.

**Results:**

The comprehensive evaluation of yield and quality showed that 4-year-old plants scored the highest at 0.5, followed by 3-year-old plants at 0.47, while 5-year-old plants scored the lowest at 0.4. Regarding the harvest time for 4-year-old plants, September 26 yielded the highest score at 0.87, followed by October 16 at 0.46, whereas September 11 received the lowest score at 0.41. Among the drying methods, shade drying resulted in the highest quality, ranking as follows: shade drying > step-down drying (105–80 °C) > high-temperature drying (>80 °C) > sun drying > low-temperature drying (60 °C). Correlation analysis revealed a significant positive correlation between paeoniflorin content and root dry weight across different harvesting ages. Additionally, paeoniflorin showed significant positive correlations with the contents of catechin, oxidized paeoniflorin, and lactiflorin across different harvesting dates.

**Conclusion:**

The present findings are specific to Tongliao City, Inner Mongolia, and are not universally applicable. In this regional context, the optimal harvest period for Paeoniae Radix Rubra falls in late September of the fourth growing year, and shade drying proves to be the most suitable post-harvest processing method.

## 1 Introduction

Paeoniae Radix Rubra was the dried root of *Paeonia lactiflora* Pall. or Paeonia veitchii Lynch., belonging to the Ranunculaceae family. It had a slightly bitter taste and a faint, aromatic fragrance. Its therapeutic effects included clearing heat, cooling the blood, promoting blood circulation, and relieving pain (1–3). The main bioactive constituents of Paeoniae Radix Rubra included total peony glycosides, flavonoids, volatile oils, tannins, phenolic acids, and other chemical compounds (4, 5). Paeoniflorin, a component of the total glycosides extracted from Paeoniae Radix Rubra, exhibited analgesic, anti-inflammatory, hypoglycemic, hypotensive, and antithrombotic effects (6). Lactiflorin demonstrated sedative, analgesic, anticonvulsant, immunomodulatory, antidepressant, and detoxifying properties (7, 8). Oxypaeoniflorin showed significant anti-inflammatory activity (9), whereas benzoylpaeoniflorin had anti-allergic effects (10). 1,2,3,4,6-Penta-O-galloyl-*β*-D-glucose was a polyphenolic tannin compound with diverse biological activities, including anticancer, antidiabetic, anti-inflammatory, and antioxidant properties (11–13). Gallic acid played an important role in protecting brain and nerve cells, and exhibited anti-inflammatory, anticancer, antioxidant, antimicrobial, and antidiabetic activities (14, 15). Catechin demonstrated antitumor effects, provided protection against cardiovascular and cerebrovascular diseases, scavenged free radicals, and possessed antioxidant and anti-radiation properties (16, 17). Benzoic acid had certain preservative effects but was also shown to be mutagenic (18–20).

In previous years, the growing demand for Paeoniae Radix Rubra coupled with the decline of its wild populations made cultivation the main source for supplying the market. However, the quality of the medicinal material was affected by multiple factors, including the cultivation duration, the timing of harvest in autumn, and the drying methods applied—all of which significantly influenced its final quality (21, 22). Most existing studies on medicinal materials focused predominantly on the relationship between harvest time and quality, with little or no consideration given to yield-related factors. Consequently, systematic research addressing the balance between harvest time, yield, and quality remained lacking. In light of this research gap, identifying the optimal harvest period and appropriate drying technique was of pivotal importance for ensuring the quality of medicinal products derived from Paeoniae Radix Rubra.

Studies indicated that the quality of Paeoniae Radix Rubra did not necessarily improve with increased cultivation duration, as it was influenced by multiple factors (23, 24). For Paeonia veitchii, although the paeoniflorin content did not peak at 5 years, this age yielded the highest production. Comprehensive evaluations suggested harvesting at 5 years (25). This study compared the quality and yield of 3-, 4-, and 5-year-old Paeoniae Radix Rubra to determine the optimal harvesting age for red peony.

The quality of Chinese herbal medicines was profoundly influenced by the developmental stage of their medicinal parts, making the appropriate harvesting period crucial for ensuring final product quality. Studies showed that the active constituents of Angelica dahurica varied significantly depending on the time of harvest. Harvesting in mid-July resulted in the highest concentration of active compounds, which was significantly greater than that obtained during spring harvesting (26). Huang et al. (27) investigated Scutellaria baicalensis harvested at different times and evaluated the content of medicinal substances based on morphological characteristics. They concluded that mid-October provided the highest overall quality in terms of appearance and baicalin content, making it the optimal harvest time. Fu (28) compared six bioactive compounds in Paeoniae Radix Rubra harvested at different periods and determined that late September to mid-October yielded the best results. Additional research indicated that the total content of monoterpenoid glycosides and phenolic compounds in Paeoniae Radix Rubra peaked in December, while the drying rate of the root was highest in mid-September. The comprehensive score of active ingredient content remained relatively high from September to October (29). However, optimal harvesting periods could vary across regions. This study aimed to identify the best harvest time for Paeoniae Radix Rubra in the Tongliao area by comparing the contents of eight bioactive compounds and evaluating yield.

Different processing methods not only affected the appearance quality of medicinal materials but also influenced their active constituents. During the drying process of Paeoniae Radix Rubra, multiple factors came into play. A study comparing freeze-drying and air-drying found that freeze-drying better preserved the paeoniflorin content in Paeoniae Radix Rubra (30). Fu et al. (31) proposed that forced-air drying at 66 °C was simple, feasible, and yielded stable quality based on comparisons of seven bioactive compounds. This study further refined that approach by employing eight bioactive compounds for comprehensive evaluation to determine the optimal drying method for Paeoniae Radix Rubra.

This study investigated how different age classes and autumn harvest periods affected the morphological characteristics and paeoniflorin content of Paeoniae Radix Rubra. Additionally, it applied various drying methods to 4-year-old Paeoniae Radix Rubra to identify the most suitable drying technique. These findings provided a scientific basis for determining optimal harvest timing and post-harvest drying methods in Paeoniae Radix Rubra production and cultivation. The conclusions of this study were only applicable to Tongliao City, Inner Mongolia, and were not universally generalizable.

## 2 Materials and methods

### 2.1 Test materials

The test materials were cultivated at the Traditional Chinese and Mongolian Medicine Base in Naiman Banner, Tongliao City, Inner Mongolia University for Nationalities. They were identified as *Paeonia lactiflora*. The experimental field has sandy loam soil with a pH of 7.65, an organic matter content of 6.42 g/kg, a hydrolyzable nitrogen content of 51.48 mg/kg, an available phosphorus content of 15.52 mg/kg, an available potassium content of 81.33 mg/kg, and a slowly available potassium content of 562.27 mg/kg. This study was conducted exclusively on Paeoniae Radix Rubra by Professor Zhang Chunhong of Baotou Medical College. Sampling was conducted on September 26, 2021, September 26, 2022, and September 26, 2023, through continuous sampling of Paeoniae Radix Rubra plants on the same plot. The target samples were 3-year-old, 4-year-old, and 5-year-old plants, respectively, which had been grown from transplanted seedlings, to determine relevant indicators for different cultivation years and harvest periods. The differences between different growth year groups were all statistically significant. In autumn 2022, 4-year-old Paeoniae Radix Rubra was sampled on September 11, September 26, and October 16 for relevant indicator measurements. Using the 4-year-old Paeoniae Radix Rubra harvested on September 26, 2022, as material, 5 different drying methods were established: air-drying in a shaded indoor area without sunlight, natural sun-drying on a sunny windowsill, drying in 60 and 80 °C ovens until constant weight, and drying in a 105 °C oven for 30 min followed by transfer to an 80 °C oven until constant weight (105–80 °C). All drying methods were carried out under controlled environmental conditions until the samples were dried to constant weight. Fifteen uniformly developed plants were selected for each treatment, with 5 plants per replicate and 3 biological replicates. Whole plants were dug up, bagged, and transported to the laboratory. They were washed under running water, drained of excess moisture, and stored for subsequent use.

### 2.2 Test methods

#### 2.2.1 Determination of Paeoniae Radix Rubra characteristics

Using an electronic balance, measure the fresh weight of roots. After drying to constant weight, measure the dry weight. Use a ruler to determine root length. Use a vernier caliper to measure the diameter of Paeoniae Radix Rubras (take the average diameter of the upper part of the 3 thickest lateral roots per plant). Calculate the dry-to-fresh ratio, i.e., the drying rate, using the fresh root weight and dry root weight.

#### 2.2.2 Determination of active ingredient content

Refer to the method in the 2020 edition of the Chinese Pharmacopeia to determine the active ingredient content of Paeoniae Radix Rubra. Employ high-performance liquid chromatography (HPLC, General Chapter 0512) for analysis. Extract dried Paeoniae Radix Rubra powder with 90% methanol using ultrasonication, then analyze using high-performance liquid chromatography (HPLC, Agilent LC-1260). Paeoniflorin (C₂₃H₂₈O₁₁) content shall not be less than 1.8%. Reference standards used: Paeoniflorin (Batch No. 23180–57-6, HPLC ≥98%), Gallic acid (Batch No. 149–91-7, HPLC ≥98%), Catechin reference standard (Batch No. 154–23-4 HPLC≥98%), Paeoniflorin reference standard (Batch No. 39011–91-1 HPLC≥98%), Paeoniflorin Reference Standard (Batch No. 39011–90-0 HPLC≥98%), 1,2,3,4,6-Penta-O-galloyl-*β*-D-glucose Reference Standard (Batch No. 14937–32-7 HPLC≥99%), Benzoic acid reference standard (Batch No. 65–85-0, HPLC ≥98%), and Benzylpaeoniflorin reference standard (Batch No. 38642–49-8, HPLC ≥98%) were all purchased from Shanghai Yuanye Biotechnology Co., Ltd.

##### 2.2.2.1 Chromatographic conditions

Column: Mobile phase: Phase A is acetonitrile; Mobile Phase: Phase A is acetonitrile; Phase B is 0.1% phosphoric acid aqueous solution. Gradient Elution: 0–10 min, 5–10% A; 20–20 min, 10–20% A; 20–30 min, 20% A; 30–40 min, 20–40% A; 40–50 min, 40–5% A. Flow Rate: 1.00 mL/min. Detection wavelength: 231 nm; Column temperature: 25 °C; Injection volume: 5 μL. Paeoniflorin eluted at 13.539 min; 1,2,3,4,6-Penta-O-galloyl-β-D-glucose eluted at 20.522 min; Lactiflorin eluted at 11.65 min; Catechin elutes at 7.45 min; Benzoylpaeoniflorin elutes at 35.713 min; Gallic acid elutes at 1.679 min; Benzoic acid elutes at 14.801 min; Oxypaeoniflorin elutes at 7.395 min.

##### 2.2.2.2 Linear regression equations

Plot a standard curve with the reference standard injection volume as the x-axis (X) and peak area as the y-axis (Y). Perform linear regression to obtain the regression equations for the 8 components: Paeoniflorin: *y* = 150.53x + 3.0219 (*R*^2^ = 0.9993), The concentration ranges from 87.2 μg/mL ~ 2.16 μg/mL.; 1,2,3,4,6-Penta-O-galloyl-β-D-glucose: *y* = 348.78x + 6.8446 (*R*^2^ = 0.9998), The concentration ranges from 93.60 μg/mL ~ 2.344 μg/mL.; Lactiflorin: *y* = 300.59x − 3.8134 (*R*^2^ = 0.9999), The concentration ranges from 206.4 μg/mL ~ 5.2 μg/mL; Catechin: *y* = 295.14x + 4.6278 (*R*^2^ = 0.997), The concentration ranges from 84.8 μg/mL ~ 2.72 μg/mL; Benzylpaeoniflorin: *y* = 231.26x − 6.3072 (*R*^2^ = 0.9998), The concentration ranges from 80.8 μg/mL ~ 2 μg/mL; Gallic acid: *y* = 394.19x + 3.9478 (*R*^2^ = 0.9997), The concentration ranges from 133.6 μg/mL ~ 4.32 μg/mL; Benzoic acid: *y* = 13,247x + 26.06 (*R*^2^ = 0.9992), The concentration ranges from 261.8 μg/mL ~ 45.9 μg/mL; Oxypaeoniflorin: *y* = 108.26x + 0.712 (*R*^2^ = 0.9996), The concentration ranges from 245.6 μg/mL ~ 6.16 μg/mL. The unit for component quantification is % (percentage).

##### 2.2.2.3 Precision test

The test solution of Paeoniae Radix Rubra was injected six times consecutively under the chromatographic conditions described in section “2.2.2.1.” The relative standard deviations (RSD) of the retention times and peak areas of each chromatographic peak were all less than 0.95%, meeting the precision error requirement.

##### 2.2.2.4 Repeatability test

For the four-year-old samples, a parallel operation was performed, and nine independent test solutions were prepared. Under the chromatographic conditions described in section “2.2.2.1,” the RSD values of the retention times of each chromatographic peak were all less than 0.13%, and the RSD values of the peak areas were all less than 3.99%, indicating that the method was feasible.

##### 2.2.2.5 Spike recovery experiment

Nine portions of 0.2 g of the sample with known active ingredient content were weighed. Reference solutions at three concentration levels (low, medium, high) were added to the portions (three portions for each level), respectively. The samples were determined according to the method, and the spike recoveries were calculated. The results ranged from 97 to 100%, indicating that the method had good recovery.

#### 2.2.3 Comprehensive index evaluation

##### 2.2.3.1 Selection of evaluation indicators

The following evaluation indicators were selected: paeoniflorin, 1,2,3,4,6-Penta-O-galloyl-β-D-glucose, Lactiflorin, catechin, Oxypaeoniflorin, gallic acid, benzoic acid, Benzoylpaeoniflorin, and root dry weight.

##### 2.2.3.2 CRITIC

Perform correlation analysis on the required evaluation indicators to obtain the correlation coefficient matrix R = (R_ij_) _13 × 13_. Calculate the weights according to the following formula (32) (1):


$$C_{j}=\mathrm{sj}\sum_{i=1}^{n}(1-R_{\mathrm{ij}}),\;(j=1,2,3,\cdots ,n)$$ (1)

In the formula, Cj represents the information contained in the jth evaluation indicator, and Rij denotes the correlation coefficient between evaluation indicators i and j. The larger the Cj is, the greater the amount of information contained in the jth evaluation indicator, and the greater its relative importance. Therefore, the objective weight of the jth evaluation indicator can be expressed as (2):


$$W_{\mathrm{ij}}=\mathrm{Cj}/\sum_{j=1}^{n}\mathrm{Cj}\;(j=1,2,3,\cdots ,n)$$ (2)

#### 2.2.4 Data analysis

Experimental data were analyzed and plotted using Excel and Origin 2024 software; variance significance analysis was performed using DPS and SPSS software.

## 3 Results and analysis

### 3.1 Changes in morphological characteristics and medicinal compounds of Paeoniae Radix Rubra harvested at different ages

**Figure 1:**
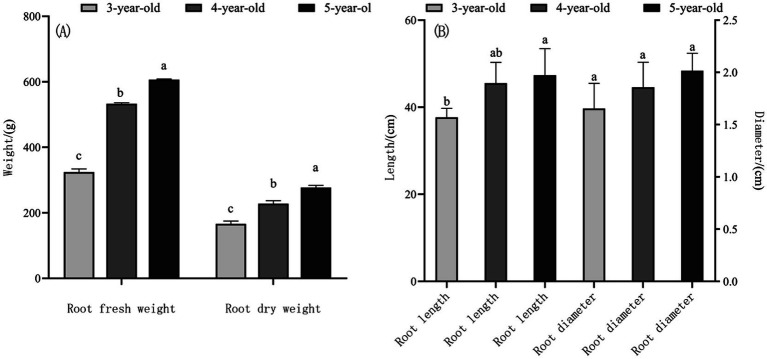
Biomass and agronomic traits of *Paeoniae Radix Rubra fro*m different cultivation years. **(A)** Root fresh and dry weight. **(B)** Root length and diameter. Different letters indicate significant differences (*p* < 0.05). The same abbreviations apply to the figures that follow.

The morphological characteristics of Paeoniae Radix Rubra harvested at different ages are shown in Figure 1. The root dry weight ranged from 165.82 to 276.96 g, showing a gradual increase with plant age. 5-year-old roots exhibited the highest fresh and dry weights, which were significantly greater than those of 4- and 3-year-old roots. The growth rates of dry weight for 4-year-old and 5-year-old roots were 37.4 and 21.56%, respectively, with the 4-year-old roots showing a growth rate 1.73 times that of the 5-year-old roots.

Root length increased with age, ranging from 37.67 to 47.33 cm. The 5-year-old roots were the longest, significantly exceeding the length of 3-year-old roots. A notable increase in root length was observed between 3 and 4 years of age, with growth rates of 20.78 and 4.02% for 4-year-old and 5-year-old roots, respectively. Root diameter ranged from 1.65 to 2.02 cm. Although there were no significant differences among the 3 age groups, an increasing trend in diameter with age was evident.

**Figure 2:**
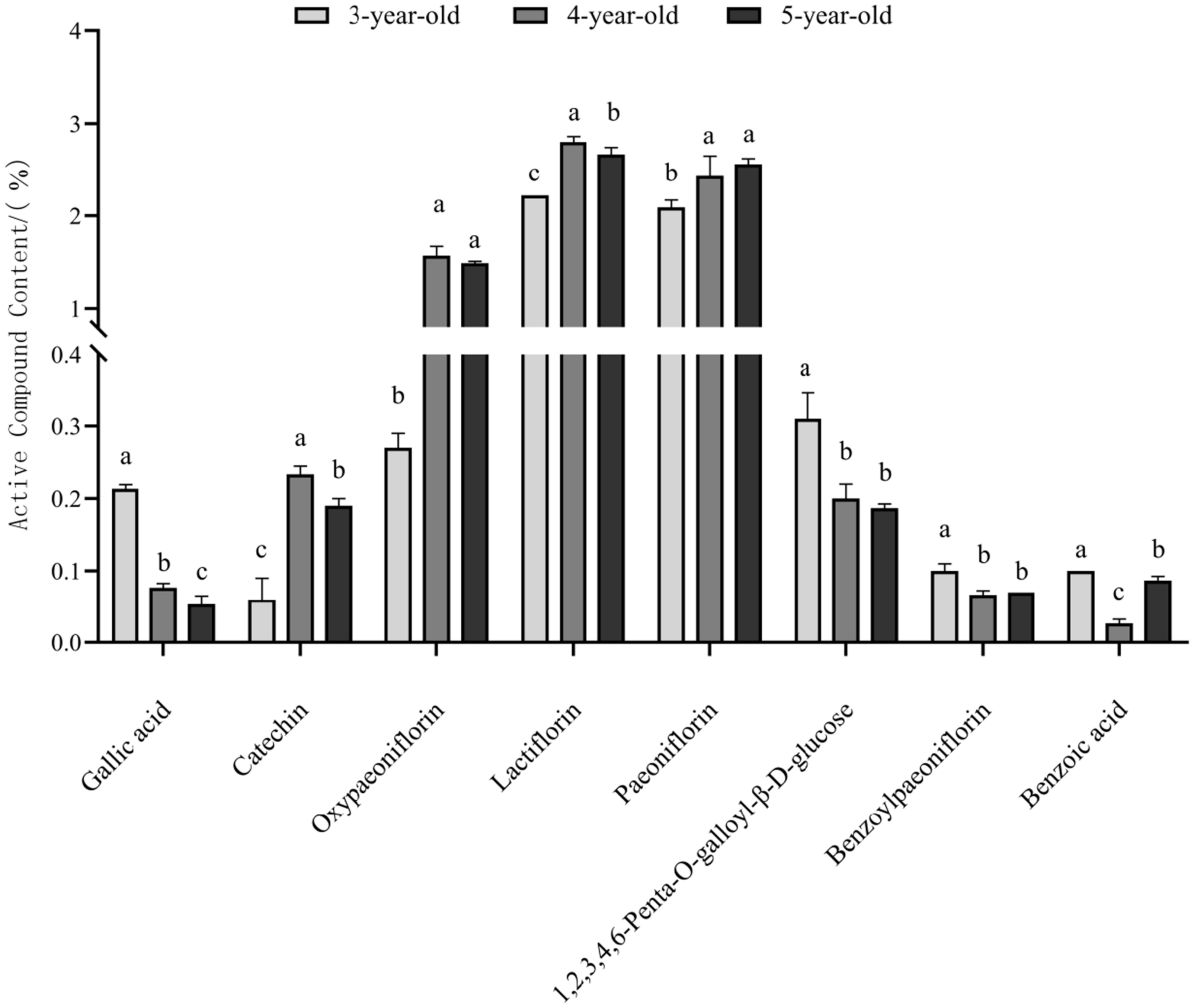
Variations of pharmacologically active compounds in *Paeoniae Radix Rubra roo*ts under different harvesting years.

The pharmacodynamic substances in Paeoniae Radix Rubra harvested at different ages are shown in Figure 2 and Table 1. The content of gallic acid, 1,2,3,4,6-Penta-O-galloyl-β-D-glucose, exhibited a gradual decreasing trend. Gallic acid showed significant differences across all 3 age groups, while 1,2,3,4,6-Penta-O-galloyl-β-D-glucose exhibited no significant differences between 4-year-old and 5-year-old plants. Catechin, Oxypaeoniflorin, and Lactiflorin exhibited an initial increase followed by a decrease. The highest levels of these 3 active compounds were found in the 4-year-old plants, followed by the 5-year-old plants. Catechin and Oxypaeoniflorin levels in the 4-year-old plants were higher than those in the 5-year-old plants., but there was no significant difference in Oxypaeoniflorin levels between the two age groups. The contents of catechin, paeoniflorin, and lactiflorin in 5-year-old plants were significantly higher than those in 3-year-old plants. Paeoniflorin showed a consistently increasing trend, with the highest content in the 5-year-old plants, followed by the 4-year-old plants. There was no significant difference between the two, but both were significantly higher than the paeoniflorin content in the 3-year-old plants. The contents of Benzoic acid and Benzoylpaeoniflorin initially decreased and then increased, with the highest content observed in the 3-year-old plants, which was significantly higher than that in the 4-year-old and 5-year-old plants.

**Table 1:** Content of active ingredients in different harvest years.

Different harvesting periods	Gallic acid(%)	Catechin(%)	Oxypaeoniflorin(%)	Lactiflori(%)	Paeoniflorin(%)	1,2,3,4,6-Penta-O-galloyl-*β*-D-glucose(%)	Benzoylpaeoniflorin(%)	Benzoic acid(%)
3-year-old	0.21 ± 0.01a	0.06 ± 0.03c	0.27 ± 0.02b	2.22 ± 0.00c	2.09 ± 0.08b	0.31 ± 0.04a	0.1 ± 0.01a	0.1 ± 0.00a
4-year-old	0.08 ± 0.01b	0.24 ± 0.01a	1.57 ± 0.1a	2.8 ± 0.06a	2.43 ± 0.22a	0.2 ± 0.02b	0.07 ± 0.01b	0.03 ± 0.00c
5-year-old	0.08 ± 0.01b	0.19 ± 0.01b	1.49 ± 0.02a	2.66 ± 0.08b	2.56 ± 0.06a	0.19 ± 0.01b	0.07 ± 0.00b	0.09 ± 0.01b

Different letters indicate significant differences (*p* < 0.05).

### 3.2 Correlation analysis between active compounds and root traits in Paeoniae Radix Rubra of different harvest years

**Figure 3:**
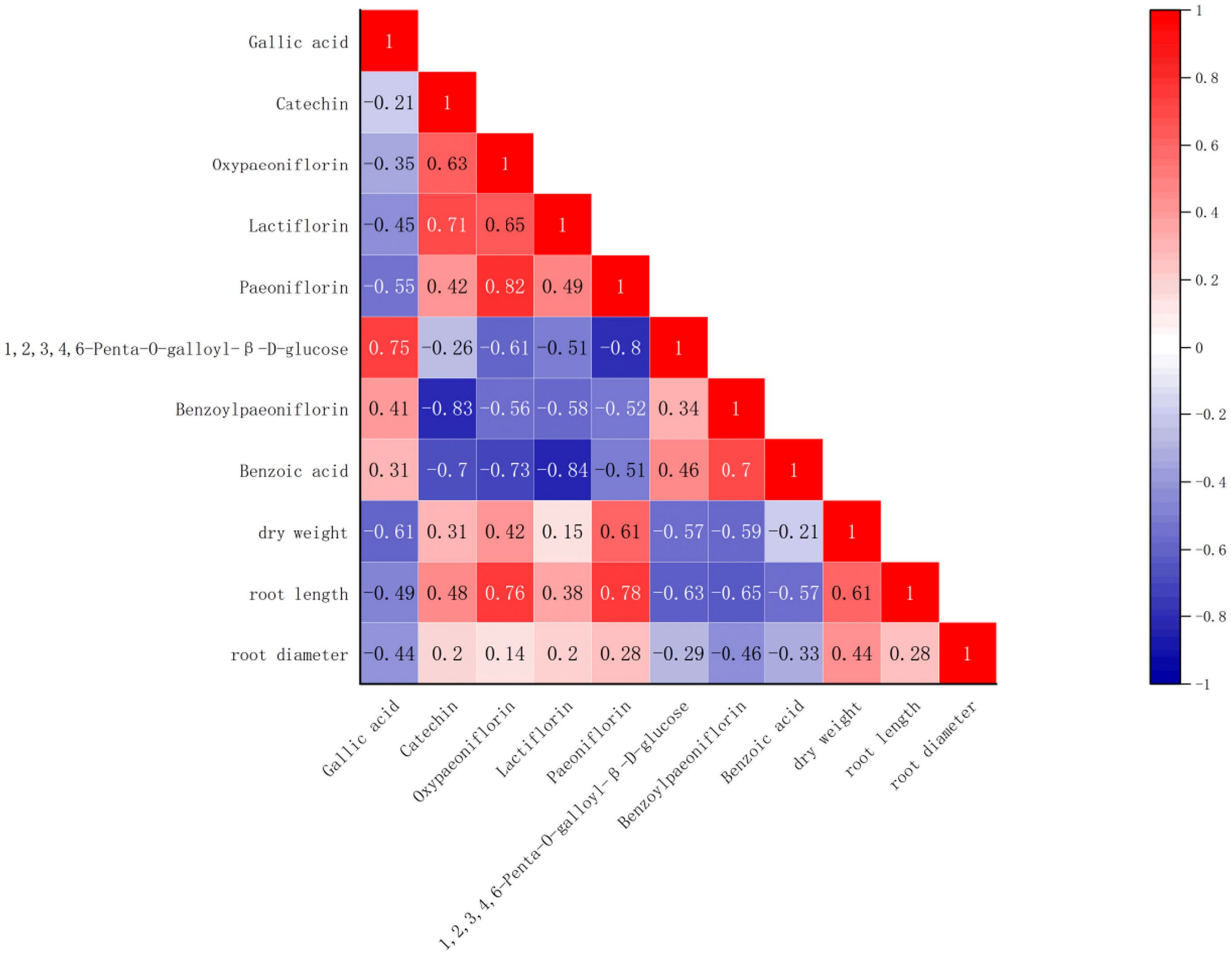
Correlation between the content of pharmacologically active compounds and root biomass in *Paeoniae Radix Rubra* across different cultivation years. Correlation analysis was performed using Kendall’s tau rank correlation coefficient. A significance level of *p* < 0.05 was used. The same applies to the subsequent figures and tables.

The correlation analysis between active compounds and root traits across different harvest years is shown in Figure 3. Root dry weight exhibited a significant negative correlation with gallic acid, benzylpaeoniflorin, and 1,2,3,4,6-Penta-O-galloyl-β-D-glucose, and a significant positive correlation with paeoniflorin. Root length showed a significant positive correlation with paeoniflorin, paeoniflorin glycoside, and dry weight, while exhibiting a significant negative correlation with paeoniflorin glycoside, benzoic acid, and 1,2,3,4,6-Penta-O-galloyl-*β*-D-glucose. Root diameter followed a similar trend to root length but did not show a significant relationship. Among the active compounds, paeoniflorin showed a significant negative correlation with gallic acid and 1,2,3,4,6-Penta-O-galloyl-β-D-glucose, and a significant positive correlation with paeoniflorin-O-acetate. Paeoniflorin showed significant negative correlations with paeoniflorin benzoate, benzoic acid, and 1,2,3,4,6-Penta-O-galloyl-β-D-glucose, and significant positive correlations with catechin and Lactiflorin. Lactone paeoniflorin showed a significant negative correlation with benzyl paeoniflorin and benzoic acid, and a significant positive correlation with catechin. Catechin showed significant negative correlations with benzylsaponin and benzoic acid. Benzylsaponin showed a significant positive correlation with benzoic acid. 1,2,3,4,6-Penta-O-galloyl-β-D-glucose showed a significant positive correlation with gallic acid.

### 3.3 Changes in bioactive compound content of Paeoniae Radix Rubra during different drying processes

**Figure 4:**
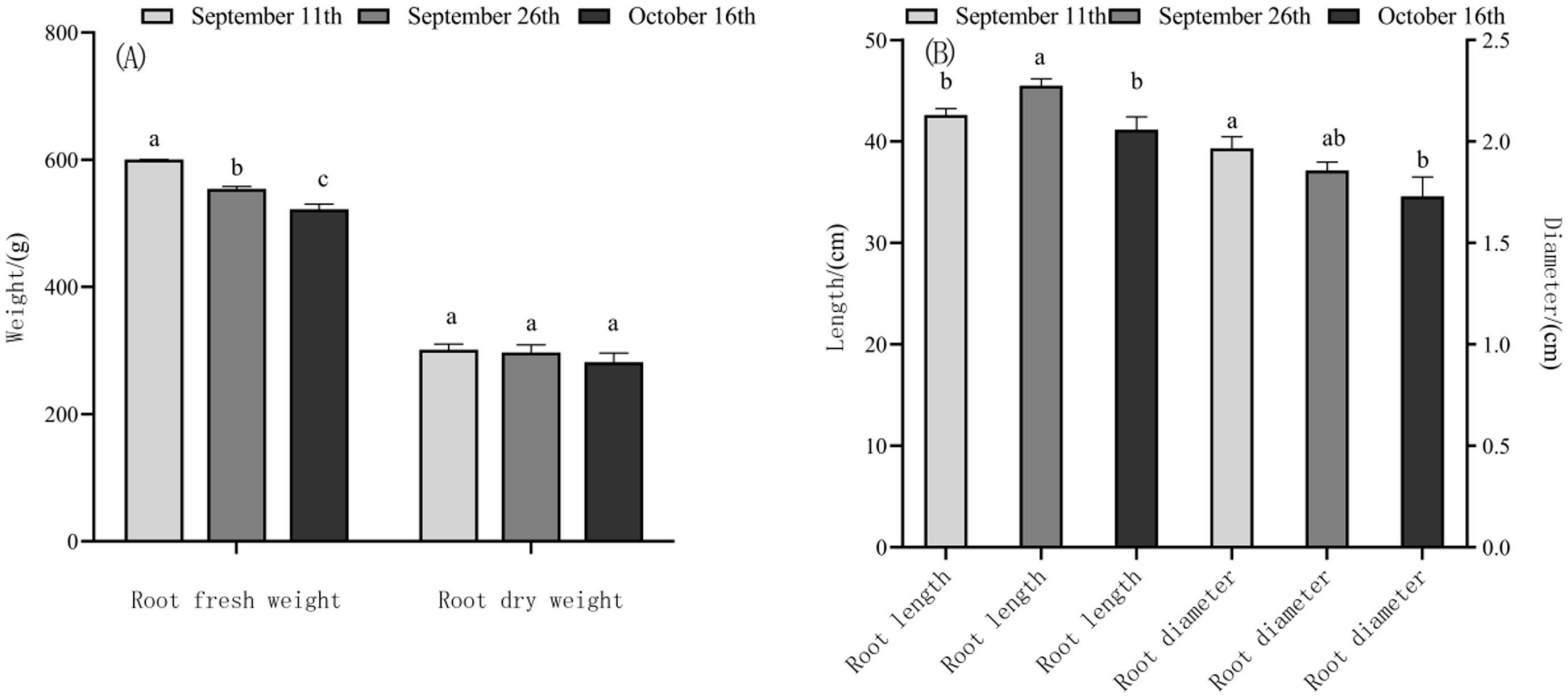
Agronomic traits of 4-year-old *Paeoniae Radix Rubra root*s at different harvesting periods. **(A)** Root fresh and dry weight. **(B)** Root length and diameter.

Changes in traits of 4-year-old Paeoniae Radix Rubra at different harvesting periods are shown in Figure 4. Fresh root weight and dry root weight exhibited a gradual decline with later harvesting periods, with single-plant dry root weight ranging from 301.10 to 281.77 g. No significant differences were observed among the 3 harvesting periods. Root length ranged from 45.5 to 41.17 cm, with roots harvested on September 26th being significantly longer than those on September 11th and October 16th. Root diameter ranged from 1.97 to 1.73 cm, peaking on September 11th and being significantly larger than on October 16th.

**Figure 5:**
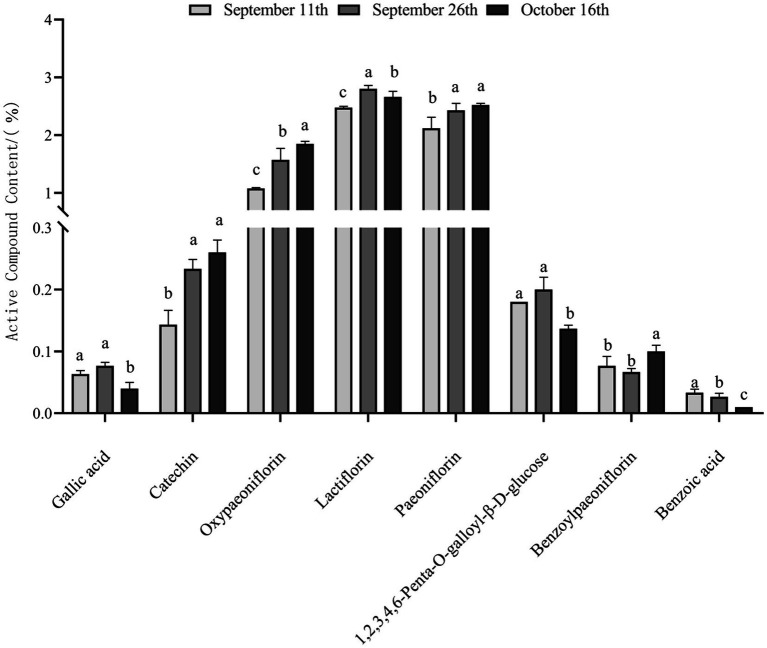
Variation of pharmacologically active compounds in 4-year-old *Paeoniae Radix Rubra roo*ts at different harvesting periods.

Changes in the content of active compounds in 4-year-old Paeoniae Radix Rubra at different harvest periods are shown in Figure 5 and Table 2. The contents of gallic acid, Lactiflorin, and 1,2,3,4,6-Penta-O-galloyl-β-D-glucose all exhibited a trend of first increasing and then decreasing, peaking on September 26. Gallic acid and 1,2,3,4,6-Penta-O-galloyl-β-D-glucose showed no significant difference between September 26 and September 11 levels, but both were significantly higher than on October 16. Paeoniflorin exhibited significant differences across the 3 harvest dates.

**Table 2:** Content of active ingredients in different harvest periods.

Different harvesting periods	Gallic acid(%)	Catechin(%)	Oxypaeoniflorin(%)	Lactiflori(%)	Paeoniflorin(%)	1,2,3,4,6-Penta-O-galloyl-β-D-glucose(%)	Benzoylpaeoniflorin(%)	Benzoic acid(%)
September 11th	0.06 ± 0.00a	0.14 ± 0.02b	1.08 ± 0.01c	2.48 ± 0.02c	2.12 ± 0.19b	0.18 ± 0.00a	0.08 ± 0.02b	0.03 ± 0.00a
September 26th	0.08 ± 0.01a	0.24 ± 0.01a	1.57 ± 0.1b	2.8 ± 0.06a	2.43 ± 0.22a	0.2 ± 0.02a	0.07 ± 0.01b	0.03 ± 0.00b
October 16th	0.04 ± 0.01b	0.26 ± 0.02a	1.85 ± 0.04a	2.66 ± 0.1b	2.52 ± 0.03a	0.14 ± 0.01b	0.1 ± 0.01a	0.01 ± 0.00c

Different letters indicate significant differences (*p* < 0.05).

Catechin, Oxypaeoniflorin, and paeoniflorin showed a gradually increasing trend. The 3 active compounds reached their highest levels on October 16. The content of paeoniflorin and catechin on October 16 did not differ significantly from that on September 26, but was significantly higher than that on September 11.

Benzoic acid exhibited a gradual decreasing trend, with significant differences among the 3 harvest periods.

Benzoylpaeoniflorin showed a trend of first decreasing and then increasing, with the highest content recorded on October 16, which was significantly higher than the other two periods.

### 3.4 Correlation analysis of pharmacologically active compounds and root traits in 4-year-old Paeoniae Radix Rubra at different harvesting periods

**Figure 6:**
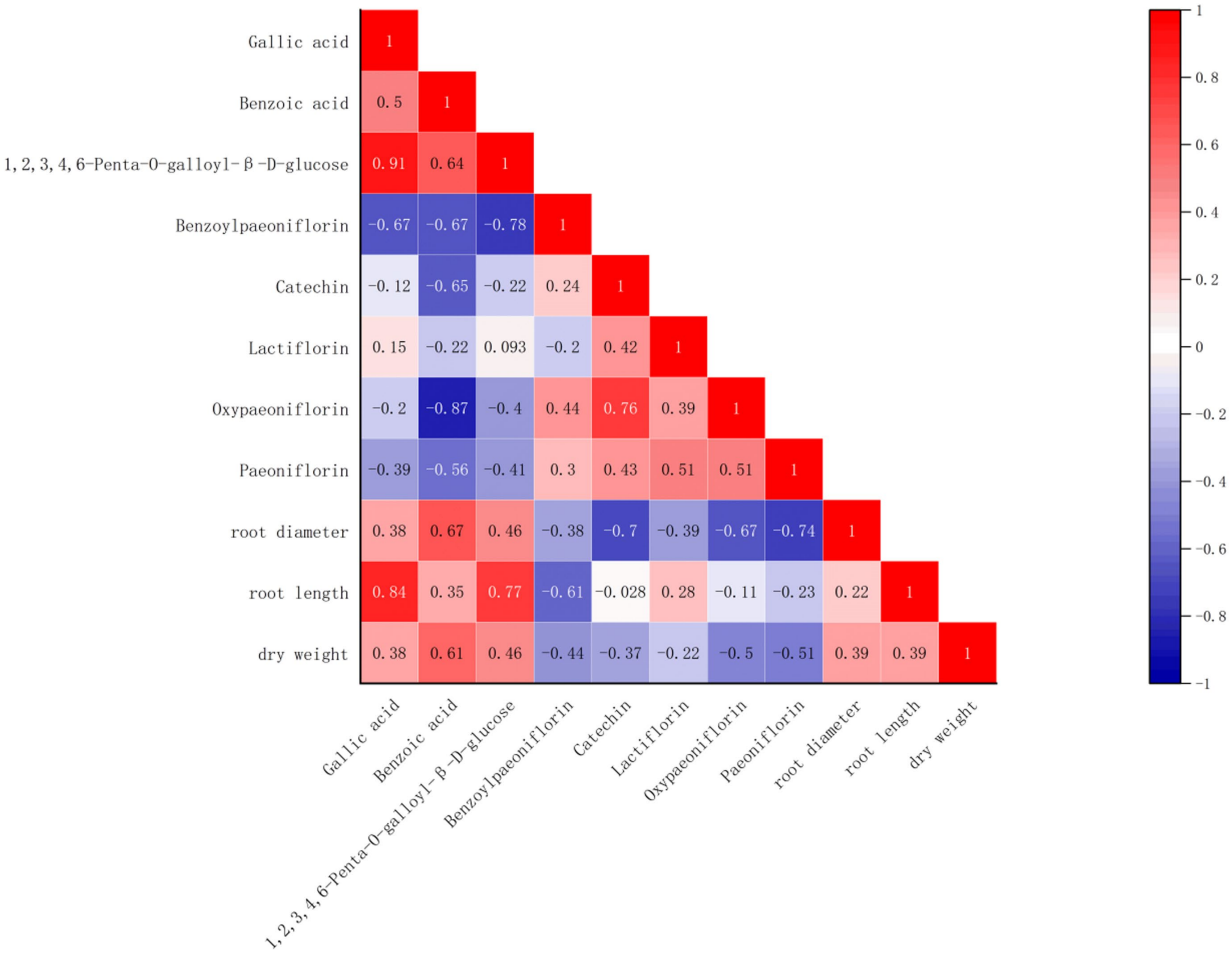
Correlation between the content of pharmacologically active compounds and root yield in *Paeoniae Radix Rubra at* different harvesting periods.

The correlation analysis between pharmacologically active compounds and yield of Paeoniae Radix Rubra at different harvesting periods is shown in Figure 6. Root dry weight exhibited a significant positive correlation with benzoic acid. Root length showed a significant negative correlation with benzylpaeoniflorin and a significant positive correlation with gallic acid and 1,2,3,4,6-Penta-O-galloyl-β-D-glucose. Root diameter exhibited a significant negative correlation with catechin, Oxypaeoniflorin, and paeoniflorin, and a significant positive correlation with benzoic acid.

Among the pharmacologically active compounds, paeoniflorin showed no significant correlations. Oxypaeoniflorin exhibited a significant positive correlation with catechin and a significant negative correlation with benzoic acid. Lactiflorin also showed no significant correlations. Catechin exhibited a significant negative correlation with benzoic acid. Benzoylpaeoniflorin showed significant negative correlations with gallic acid, benzoic acid, and 1,2,3,4,6-Penta-O-galloyl-β-D-glucose. 1,2,3,4,6-Penta-O-galloyl-β-D-glucose exhibited significant positive correlations with gallic acid and benzoic acid.

### 3.5 Changes in bioactive compound content of Paeoniae Radix Rubra during different drying methods

**Figure 7:**
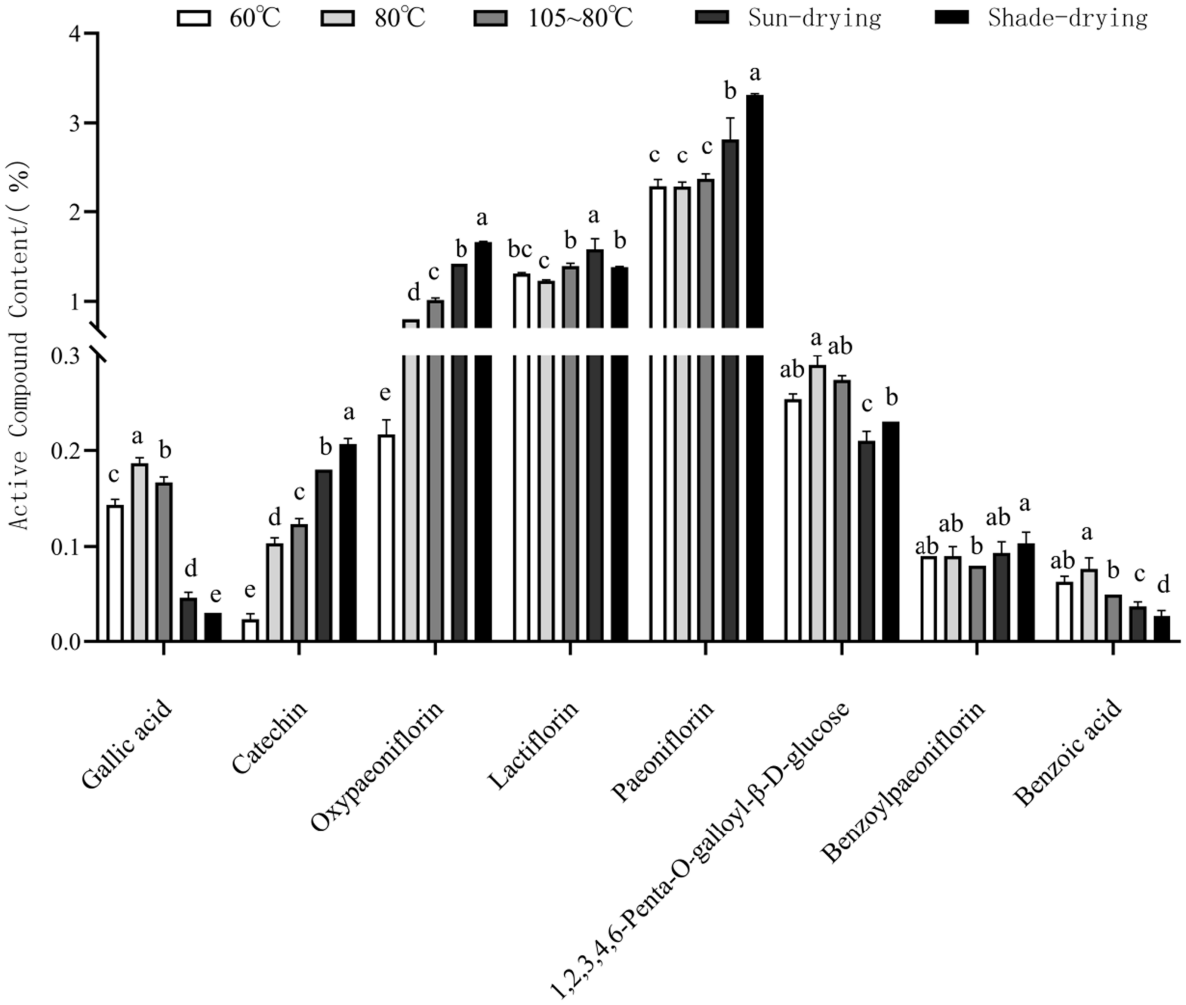
Content of pharmacologically active compounds in *Paeoniae Radix Rubra roo*ts under different drying methods.

The content of bioactive compounds in Paeoniae Radix Rubra roots subjected to different drying methods is shown in Figure 7 and Table 3. When dried at 60 °C, except for 1,2,3,4,6-Penta-O-galloyl-β-D-glucose, paeoniflorin, and benzoic acid—which showed relatively high content—the remaining 5 bioactive compounds were present at low levels. At 80 °C, gallic acid, benzoic acid, and 1,2,3,4,6-Penta-O-galloyl-β-D-glucose exhibited the highest concentrations, with benzoylpaeoniflorin also being relatively high. The other four compounds showed lower values. Step-down drying from 105 to 80 °C resulted in moderate levels of catechin, oxypaeoniflorin, and benzoic acid, whereas paeoniflorin and benzoylpaeoniflorin were low. In contrast, 1,2,3,4,6-Penta-O-galloyl-β-D-glucose, lactiflorin, and gallic acid were relatively high. Sun-drying led to moderate concentrations of catechin; paeoniflorin and benzoylpaeoniflorin were relatively high—second only to the highest values observed among the methods. The contents of 1,2,3,4,6-Penta-O-galloyl-β-D-glucose, gallic acid, and benzoic acid were relatively low, while lactiflorin reached its highest content under this condition. Shade drying yielded the highest contents of catechin, paeoniflorin, and benzoylpaeoniflorin, which were significantly greater than those under the other 4 drying methods. The contents of 1,2,3,4,6-Penta-O-galloyl-β-D-glucose and lactiflorin were relatively low, while gallic acid and benzoic acid showed the lowest levels among the 5 methods.

**Table 3:** Content of active ingredients in different drying methods.

Different drying methods	Gallic acid (%)	Catechin (%)	Oxypaeoniflorin (%)	Lactiflori (%)	Paeoniflorin (%)	1,2,3,4,6-Penta-O-galloyl-β-D-glucose (%)	Benzoylpaeoniflorin (%)	Benzoic acid (%)
60 °C	014 ± 0.00c	0.02 ± 0.01e	0.22 ± 0.01e	1.31 ± 0.01bc	2.28 ± 0.08c	0.26 ± 0.03ab	0.09 ± 0.00ab	0.06 ± 0.01ab
80 °C	0.19 ± 0.00a	0.10 ± 0.01d	0.8 ± 0.00d	1.23 ± 0.01c	2.28 ± 0.05c	0.29 ± 0.02a	0.09 ± 0.01ab	0.08 ± 0.01a
105 ~ 80 °C	0.17 ± 0.01b	0.12 ± 0.00c	1.01 ± 0.0ec	1.39 ± 0.03b	2.37 ± 0.06c	0.27 ± 0.03ab	0.08 ± 0.00b	0.05 ± 0.00b
Sun-drying	0.04 ± 0.00d	0.18 ± 0.00b	1.42 ± 0.00b	1.58 ± 0.12a	2.82 ± 0.24b	0.21 ± 0.02c	0.09 ± 0.01ab	0.04 ± 0.00c
Shade-dryin	0.03 ± 0.00e	0.21 ± 0.00a	1.66 ± 0.01a	1.38 ± 0.01b	3.31 ± 0.02a	0.23 ± 0.00b	0.1 ± 0.01a	0.02 ± 0.01d

Different letters indicate significant differences (*p* < 0.05).

### 3.6 Comprehensive evaluation of red peony yield and quality using the CRITIC method

Evaluation indicators included paeoniflorin, 1,2,3,4,6-Penta-O-galloyl-β-D-glucose, Lactiflorin, catechin, benzylpaeoniflorin, gallic acid, benzoic acid, oxidized paeoniflorin, and root dry weight. The CRITIC weighting method was applied to analyze the weight composition of each indicator across different age-class Paeoniae Radix Rubra datasets and harvest years, yielding the composite scores shown in Tables 4, 7. As Table 7 indicates, among the composite scores for different harvest years, the 4-year-old plants achieved the highest score (0.50), followed by the 3-year-old plants (0.47), while the 5-year-old plants scored lowest (0.40). Thus, the 4-year-old plants were determined to be optimal. The comprehensive scores for different harvest periods are shown in Tables 5, 7. As indicated in Table 7, the highest score was achieved on September 26 (0.87), followed by October 16 (0.46), with the lowest score on September 11 (0.41). Thus, September 26 is the optimal harvest date. The comprehensive scores for different drying methods are shown in Tables 6, 7. As indicated in Table 7, air-drying achieved the highest comprehensive score, followed by shade drying > step-down drying (105–80 °C) > high-temperature drying (>80 °C) > sun drying > low-temperature drying (60 °C) (Tables 5–7).

**Table 4:** Calculation results of CRITIC weights for different years.

Item	Indicator contradiction	Indicator variability	Information amount	Weight%
NMMS_Benzoic acid	0.541	6.816	3.686	9.2010
MMS_Gallic acid	0.532	11.229	5.969	14.8977
MMS_Catechin	0.516	6.426	3.317	8.2791
MMS_Oxypaeoniflorin	0.560	6.531	3.660	9.1353
MMS_Lactiflorin	0.522	6.436	3.358	8.3822
MMS_Paeoniflorin	0.516	6.903	3.565	8.8968
MMS_1,2,3,4,6-Penta-O-galloyl-β-D-glucose	0.555	11.350	6.298	15.7188
MMS_Benzoylpaeoniflorin	0.577	11.424	6.596	16.4621
MMS_Dry weight	0.501	7.217	3.617	9.0270

**Table 5:** Calculation results of CRITIC weights for different harvest periods.

Item	Indicator contradiction	Indicator variability	Information amount	Weight%
NMMS_Benzoic acid	0.509	5.219	2.658	8.8338
MMS_Gallic acid	0.500	4.716	2.358	7.8383
MMS_Catechin	0.536	7.439	3.986	13.2483
MMS_Oxypaeoniflorin	0.506	8.435	4.269	14.1913
MMS_Lactiflorin	0.501	5.102	2.558	8.5020
MMS_Paeoniflorin	0.525	7.717	4.048	13.4561
MMS_1,2,3,4,6-Penta-O-galloyl-β-D-glucose	0.577	6.417	3.705	12.3154
MMS_Benzoylpaeoniflorin	0.509	5.219	2.658	8.8338
MMS_Dry weight	0.527	7.302	3.845	12.7811

**Table 6:** Calculation results of CRITIC weights for different drying methods.

Item	Indicator contradiction	Indicator variability	Information amount	Weight%
NMMS_Benzoic acid	0.373	5.046	1.880	9.0395
MMS_Gallic acid	0.465	10.620	4.936	23.7271
MMS_Catechin	0.373	4.597	1.714	8.2410
MMS_Oxypaeoniflorin	0.377	4.603	1.736	8.3458
MMS_Lactiflorin	0.371	6.488	2.408	11.5742
MMS_Paeoniflorin	0.437	4.717	2.060	9.9030
MMS_1,2,3,4,6-Penta-O-galloyl-β-D-glucose	0.399	10.098	4.031	19.3795
MMS_Benzoylpaeoniflorin	0.365	5.577	2.037	9.7900

**Table 7:** Comprehensive scores of different treatments.

Treatment conditions	Different treatments	Comprehensive score	Score ranking
Different year intervals	3-year-old	0.47	2
	4-year-old	0.50	1
	5-year-old	0.40	3
Different harvest times	Sep 11	0.41	3
	Sep 26	0.87	1
	Oct 16	0.46	2
Different drying methods	60 °C	0.37	5
	80 °C	0.53	3
	80 °C ~ 105 °C	0.54	2
	Sun-drying	0.40	4
	Shade-drying	0.55	1

## 4 Discussion

As a perennial root medicinal material, the harvesting period was a key factor in ensuring the quality of Paeoniae Radix Rubra. The duration of growth significantly impacted both yield and medicinal efficacy (21). Research indicated that 3-year-old Paeoniae Radix Rubra from Bozhou, Anhui Province exhibited the highest comprehensive content of medicinal substances (33). A comparative study of 1-, 4-, and 7-year-old roots found that the primary active components reached relatively high levels in 4-year-old roots, with minimal further increase in 7-year-old roots (34). At experimental sites, 5-year-old Paeoniae Radix Rubra frequently exhibited diseases. Kang Xiaofei’s (35) research identified that 5-year-old and older Paeoniae Radix Rubra roots were prone to root rot and other diseases. Wang and Shi (36) observed hollowed-out and lignified roots in 6-year-old Paeoniae Radix Rubra, which degraded medicinal quality. Herbal material prices fluctuated significantly and were influenced by uncertain factors. Therefore, the optimal harvest timing for red peony should be determined by comprehensively considering the growth characteristics of the herb, market demand, and time costs (37). While the yield of medicinal materials was an important indicator of their economic value, quality remained the core determinant, as high-quality materials could generate greater value (38). In this study, 5-year-old Paeoniae Radix Rubra exhibited a high yield, but its susceptibility to diseases such as root rot resulted in reduced quality. Studies showed that the susceptibility of Paeonia veitchii to root rot was associated with its perennial growth habit and soil acidification caused by continuous cropping (39). Furthermore, for 5-year-old plants, the longer growth period led to a year-by-year increase in disease incidence (40).

Without adequate quality support, a high yield alone could not achieve the expected economic and medicinal value. Therefore, in the CRITIC method, the weight assigned to quality should be much higher than that assigned to yield (41, 42). Consequently, although 5-year-old Paeoniae Radix Rubra had the highest yield, it received the lowest comprehensive score.

In the correlation analysis between active compounds and yield across different harvest years, root dry weight, root diameter, and root length showed consistent trends with active compounds. Among the active compounds, paeoniflorin exhibited significant negative correlations with gallic acid and 1,2,3,4,6-penta-O-galloyl-β-D-glucose. Research indicated that Paeoniae Radix Rubra with higher paeoniflorin content often contained lower levels of cinnamic acid and its derivatives (43). The role of paeoniflorin in enhancing insulin sensitivity and regulating glucose metabolism might lead to reduced levels of certain glucose-related compounds, potentially including 1,2,3,4,6-penta-O-galloyl-β-D-glucose (44). Paeoniflorin showed a significant positive correlation with oxidized paeoniflorin, possibly because metabolic products of paeoniflorin, including its oxidized form (45), might interact through similar cellular pathways, leading to their positive correlation (46).

Correlations between active compounds and yield of Paeoniae Radix Rubra at different harvest periods: root dry weight and root length were positively correlated with benzoic acid. Nutrient concentration variations affected root diameter, and benzoic acid might influence root traits similarly (47). Correlation trends with paeoniflorin and other active compounds generally aligned across different age groups, but benzoic acid and oxypaeoniflorin exhibited opposite trends, warranting further investigation.

Pharmacopeia records indicated spring and autumn as optimal harvest seasons. In a comparative study by Jian et al. (34) between autumn and spring harvests, autumn was proposed as the optimal harvesting period. Therefore, considering the balance of active compounds, biomass accumulation, and practical production, late September was determined as the optimal harvest period for four-year-old plants. This conclusion aligned with the findings of Fu et al. (28) and Miao et al. (48).

Different drying methods for medicinal materials also significantly impacted their quality. Shade-dried Paeoniae Radix Rubra contained the highest paeoniflorin content, followed by sun-dried material. Research indicated that prolonged exposure to sunlight and ultraviolet radiation significantly reduced internal paeoniflorin levels (22), necessitating avoidance of direct sunlight in practical production.

## 5 Conclusion

This study determined that 4-year-old Paeoniae Radix Rubra was the optimal harvest age by comparing the yield and quality of 3-, 4-, and 5-year-old plants, using an objective evaluation based on the CRITIC method. On this basis, a comparative experiment was conducted with 4-year-old Paeoniae Radix Rubra harvested on three dates in autumn: September 11, September 26, and October 16. The results showed variations in yield and quality across these harvest dates; comprehensive scoring using the CRITIC method identified September 26 as the optimal harvest date. Furthermore, an objective evaluation of biomass and quality under different drying methods using the CRITIC method revealed that shade-drying achieved the highest comprehensive score, with the order being shade-drying > drying at 105–80 °C > drying at >80 °C > sun-drying > drying at 60 °C, indicating that shade-drying was the most suitable method for Paeoniae Radix Rubra. In summary, the optimal harvest period for Tongliao, Inner Mongolia, China, was late September for 4-year-old plants, with shade drying being the preferred drying method.

## Data Availability

The original contributions presented in the study are included in the article/supplementary material, further inquiries can be directed to the corresponding author.
